# Supplementary material for: Adaptation of Prokaryotic Toxins for Negative Selection and Cloning-Independent Markerless Mutagenesis in Streptococcus Species
Source: mSphere. 2023 Apr 24;8(3):e00682-22. doi: 10.1128/msphere.00682-22 (PMC10286703; doi:10.1128/msphere.00682-22)
Supplement: TABLE S1 [file msphere.00682-22-s0001.docx]

| Table S1.  **Primers used in this study** |  |
| --- | --- |
| **Name of primer** | **Sequence (5′→3′)** |
| 1925-1 brsRM159-LF | CTTACCGCGACTAACATCAGCC |
| 1925-1 Rvs | GAT GTG CCT CCA TTA AAG ATT TAA |
| 1925-2 Fwd | TTA AAT CTT TAA TGG AGG CAC ATC ATCTCTTGCCAGTCACGTTAC |
| 1925-2 Rvs | ACGATATATATAAAGAAATTATGCCACATATTTACCTCCTTTGATTTAAGTGAACAAG |
| 1925-3 Fst-sm Fwd | ATGTGGCATAATTTCTTTATATATATCGT |
| 1925-3 Fst-sm Rvs | TTAATCGTCGTCTTTCTTATCCAG |
| 1925-4 Fwd | CTGGATAAGAAAGACGACGATTAAAAACAATGAATAGGTTTACACTTACTTTAG |
| 1925-IFDC-RVs | GGAGTTAGTAGGCAGTATCTTAATCGG |
| 2018-2 Fwd | CCGATTAAGATACTGCCTACTAACTCCGTTTTATTGGTATAGGAATCGGTTCTTTAATG |
| 1925-5 brsRM159-RR | TGGTTGTTCATTGTTCCCATTGCT |
| 1918-2 seq-Fwd | TAAACTTAATTTAGTTTATTTATAGATTTCATTGGC |
| 1918-5 seq-Fwd | GCAGTGTATTTACTTGAGAGGAGAC |
| 1918-5 seq-RVs | GTTCGTATGTATTCAAATATATCCTCCTCAC |
| 1925-IFDC-Fwd | ATCTCTTGCCAGTCACGTTACGTTATTAG |
| 2019 Xy1R seq Rve | TTG TAA CCC ATA TGC CTG AGT C |
| 2019-2 seq Rvs | GAT GGG TGA TTT TGA TTT TAG TAG CTA TT |
| 1915-2M Rvs | CCCTTGCTTGATGGTTACCATATTTACCTCCTTTGATTTAAGTGAACAAG |
| 1915-3 Maz Fwd | ATGGTAACCATCAAGCAAGGG |
| 1915-3 Maz Rvs | TTATCCTTTTTCAAATAGCACCTTGACC |
| 1915-4M Fwd | GGTCAAGGTGCTATTTGAAAAAGGATAAAAACAATGAATAGGTTTACACTTACTTTAG |
| 1915-2S Rvs | CTTGTTTATTTGACTTTTCCATTTTCATATTTACCTCCTTTGATTTAAGTGAACAAG |
| 1915-3 Smut Fwd | ATGAAAATGGAAAAGTCAAATAAACAAG |
| 1915-3 Smut Rvs | TCATTTGTCTGCTTCTTTCTTATCAA |
| 1915-4S Fwd | TTGATAAGAAAGAAGCAGACAAATGAAAACAATGAATAGGTTTACACTTACTTTAG |
| 1917-2R Rvs | CCATCTGTAAAAATTTCAACTTGTTTAAGCATATTTACCTCCTTTGATTTAAGTGAACAAG |
| 1917-3 RnaseH Fwd | ATGCTTAAACAAGTTGAAATTTTTACAGATGG |
| 1917-3 RnaseH Rvs | TTA AACTTCAACTTGATAACCTGTATCTTC |
| 1917-4R Fwd | GAAGATACAGGTTATCAAGTTGAAGTTTAAAAACAATGAATAGGTTTACACTTACTTTAG |
| 2019-1 Rvs | ATTTACCTCCTTTGATTTAAGTGAACAAG |
| 2019-2 ORF5-Fwd | CTT GTT CAC TTA AAT CAA AGG AGG TAA ATATGCCGATGAGGTTTTTGC |
| 2019-2 ORF5-Rvs | GAT GGG TGA TTT TGA TTT TAG TAG CTA TT |
| 2019-3 Fwd | AATAGCTACTAAAATCAAAATCACCCATCAAACAATGAATAGGTTTACACTTACTTTAGT |
| 2110-1-LF | GAGTATGATGGTAGAAGAGGCTG |
| 2110-1-LR | ACTACAAATATTCCTGCAAAGCC |
| 2110-2 ∆spxB Fwd | GGCTTTGCAGGAATATTTGTAGTATCTCTTGCCAGTCACGTTACGT TATTAG |
| 2110-2 ∆spxB Rvs | GACGTTTACCAAGTTTGTAAGGATGGAGTTAGTAGGCAGTATCTTAATCGG |
| 2110-3-RF | ATCCTTACAAACTTGGTAAACGTC |
| 2110-3-RR | CTATCCTCCATAAAAAGACCGGA |
| 2113-1-Rvs | TCATTGTTTGCCTCCCTGCTCCATCAAACTTGCATCTGCCT |
| 2021-17-1 Rvs | AGCCATTCTCTAAACATCTCCTTCAGAGGAATTATTTAATTGCGCGTGA |
| 2117-2 Fwd renG | GAAGGAGATGTTTAGAGAATGGCT |
| 2117-2 Rvs renG | TTAGATAGAACGTTGCTCATTCTTC |
| 2121-17-3 Fwd | GAAGAATGAGCAACGTTCTATCTAATCGCCGAAAATCAAATGTAAACTGT |
| 2021-17-3 Rvs | AAGCTATACAGGACAAAGTATACTGT |
| 2021-22-1 LF | GGATTGAGCATCTCTGAGCA |
| 2021-22-1 ∆spxB-Rvs | CTAATAACGTAACGTGACTGGCAAGAGATAATGGGAAGTTTGAACCAAGGAA |
| 2121-22-3-∆spxB Fwd | CCGATTAAGATACTGCCTACTAACTCCCCCTTACAAACTTGGTAAACGTC |
| 2121-22-3-RR | TCCTCCAGAAAAAGACCGGA |
| 2021-22-4 nest Fwd | AGCGTTTTCATTCACAAATGGAAATG |
| 2021-22-4 nest Rvs | CAAGGAATCGAATCACAGGCA |
| 2021-23-1 Rvs | AGCCATTCTCTAAACATCTCCTTCAGTTTACATTTGATTTTCGACGAGAAGAA |
| 2021-23-3 Fwd | GAAGAATGAGCAACGTTCTATCTAAGTGTCATCTTCACTTTGCCGTA |
| 2021-23-3 Rvs | CCTCCTAGAAAAAGAGAGAGGCT |
| 2130-1 Fwd LAM | AGC GAT TCT GAT TGA CAG TTT GA |
| 2130-1 Rvs LAM | CTAATAACGTAACGTGACTGGCAAGAGATAGGCAACGGTTTCCAGAGT |
| 2130-3 Fwd RAM | CCGATTAAGATACTGCCTACTAACTCCGAGAAACTTTTGCTAATTTTGCAATGT |
| 2130-3 Rvs RAM | CTTGAGAGAACACGACTGC |
| 2130-4 nest Fwd | GTCTGTTGAGGATATCAACAGCAG |
| 2130-4 nest Rvs | ACTCTTCAGCCATAATCCCAATC |
| 2130-5 Rvs galk mut | CCAAAAACGTGAGTAAAGGCTTAGTTTAATTCTTGTTTTTTCATAAGAATCCTT |
| 2130-6 Fwd galk mut | **T**AAGCCTTTACTCACGTTTTTGG |
| 2201-1 Fwd LAM | AGGATTTGGAGGCTCTGACA |
| 2201-1 Rvs LAM | CTAATAACGTAACGTGACTGGCAAGAGATTGGGCTATACTAAGTACCTCAAGAC |
| 2201-3 Fwd RAM | CCGATTAAGATACTGCCTACTAACTCCCATTTCTTCAGCGAAAGTTAAAATACTGT |
| 2201-3 Rvs RAM | CTT TGAA AGA TTC CAC AGC ATC CT |
| 2201-4 nest Fwd | ACCAAAGAAGCAGTGCGT |
| 2201-4 nest Rvs | CCAGTCACTTCATAGTCATGTTCC |
| 2201-5 Rvs galk mut | GCAAAATCTGCGCGGACTTACTCCGCTGAAAGTCTTTTTGACA |
| 2201-6 Fwd galk mut | TAAGTCCGCGCAGATTTTGC |
| 2131-1 Fwd LAM | AGCGACCACTATTCAGTGGA |
| 2131-1 Rvs LAM | CTAATAACGTAACGTGACTGGCAAGAGATAAGTAATGCGCCTTGTTTAGGT |
| 2131-3 Fwd RAM | CCGATTAAGATACTGCCTACTAACTCCTGATGCTGAGACGGAAGACA |
| 2131-3 Rvs RAM | AGTCTAGCACTCGGCTTCC |
| 2131-4 nest Fwd | TCGTCTTTGTTGGTCCGTC |
| 2131-4 nest Rvs | TTTGAGACGATTTTCGTCCTCT |
| 2131-6 Fwd galk mut | TAAGTCCGCGCAGATTTTGC |
