# Supplementary material for: Adaptation of Prokaryotic Toxins for Negative Selection and Cloning-Independent Markerless Mutagenesis in Streptococcus Species
Source: mSphere. 2023 Apr 24;8(3):e00682-22. doi: 10.1128/msphere.00682-22 (PMC10286703; doi:10.1128/msphere.00682-22)
Supplement: TABLE S2 [file msphere.00682-22-s0002.docx]

Table S2.

| **Plasmids and strains used in this study** | |
| --- | --- |
| **Name of plasmid or strain** | **Relevant characteristics** |
| pZX9 | plasmid containing Xyl-S cassette |
| UA159 | Wild-type *Streptococcus mutans* |
| UA140 | Wild-type *Streptococcus mutans* |
| SK36 | Wild-type *Streptococcus sanguinis* |
| DL1 | Wild-type *Streptococcus gordonii* |
| CL1 | Wild-type *Streptococcus mutans* |
| JF243 | Wild-type *Streptococcus mutans* |
| ifdLRS/brsRM-gusA | UA140:: Ø(*brsRM-gusA*), ΔSMU_294/295, ΔSMU_433/434, ΔSMU_1070c/1069c, Δ*hdrRM* |
| 159MfstS | UA159 Δ*brsM*::Ø(IFDC4-*fst-sm*A7S), Spec^R^ |
| 159MfstT | UA159 Δ*brsM*::Ø(IFDC4-*fst-sm*A7T), Spec^R^ |
| 159MparE1 | UA159 Δ*brsM*::Ø(IFDC4-*parE* RBS), Spec^R^ |
| 159MparE2 | UA159 Δ*brsM*::Ø(IFDC4-*parE* S56G), Spec^R^ |
| 159MmazF | UA159 Δ*brsM*::Ø(IFDC4-*mazF*), Spec^R^ |
| 159MsmuT | UA159 Δ*brsM*::Ø(IFDC4-*smuT*), Spec^R^ |
| 159MrnH | UA159 Δ*brsM*::Ø(IFDC4-*rnH*), Spec^R^ |
| 140MfstS | UA140 Δ*brsM*::Ø(IFDC4-*fst-sm*A7S), Spec^R^ |
| 140MfstT | UA140 Δ*brsM*::Ø(IFDC4-*fst-sm*A7T), Spec^R^ |
| 140MparE1 | UA140 Δ*brsM*::Ø(IFDC4-*parE* RBS), Spec^R^ |
| 140MparE2 | UA140 Δ*brs*M::Ø(IFDC4-*parE* S56G), Spec^R^ |
| SK36SBfstS | SK36 Δ*spxB*::(IFDC4-*fst-sm*A7S), Spec^R^ |
| SK36SBfstT | SK36 Δ*spxB*::(IFDC4-*fst-sm*A7T), Spec^R^ |
| SK36SBparE1 | SK36 Δ*spxB*::(IFDC4-*parE* RBS), Spec^R^ |
| SK36SBparE2 | SK36 Δ*spxB*::(IFDC4-*parE* S56G), Spec^R^ |
| SK36RGfstT | SK36Δ*spxB*(*fst-sm*A7T)::Ø(*spxB-renG*) |
| DL1SBfstS | DL1 Δ*spxB*::(IFDC4-*fst-sm*A7S), Spec^R^ |
| DL1SBfstT | DL1Δ*spxB*::(IFDC4-*fst-sm*A7T), Spec^R^ |
| DL1SBparE1 | DL1Δ*spxB*::(IFDC4-*parE* RBS), Spec^R^ |
| DL1SBparE2 | DL1 Δ*spxB*::(IFDC4-*parE* S56G), Spec^R^ |
| DL1RGfstT | DL1Δ*spxB*(*fst-sm*A7T)::Ø(*spxB-renG*) |
| 159GKfstT | UA159 Δ*galK*::Ø(IFDC4-*fst-sm*A7T), Spec^R^ |
| 159galk 22 | point mutation in *galK* codon 8, CAA to TAA |
| DL1GKfstT | DL1 Δ*galK*::Ø(IFDC4-*fst-sm*A7T), Spec^R^ |
| DL1galk25 | point mutation in *galK* codon 9, CAA to TAA |
| CL1MfstS | CL1 Δ*brsM*::Ø(IFDC4-*fst-sm*A7S), Spec^R^ |
| CL1MfstT | CL1 Δ*brsM*::Ø(IFDC4-*fst-sm*A7T), Spec^R^ |
| CL1MparE1 | CL1 Δ*brsM*::Ø(IFDC4-*parE* RBS), Spec^R^ |
| CL1MparE2 | CL1 Δ*brsM*::Ø(IFDC4-*parE* S56G), Spec^R^ |
| JF243MfstS | JF243 Δ*brsM*::Ø(IFDC4-*fst-sm*A7S), Spec^R^ |
| JF243MfstT | JF243 Δ*brsM*::Ø(IFDC4-*fst-sm*A7T), Spec^R^ |
| JF243MparE1 | JF243 Δ*brsM*::Ø(IFDC4-*parE* RBS), Spec^R^ |
| JF243MparE2 | JF243 Δ*brsM*::Ø(IFDC4-*parE* S56G), Spec^R^ |
