# Supplementary figures and images for: Adaptation of Prokaryotic Toxins for Negative Selection and Cloning-Independent Markerless Mutagenesis in Streptococcus Species
Source: mSphere. 2023 Apr 24;8(3):e00682-22. doi: 10.1128/msphere.00682-22 (PMC10286703; doi:10.1128/msphere.00682-22)

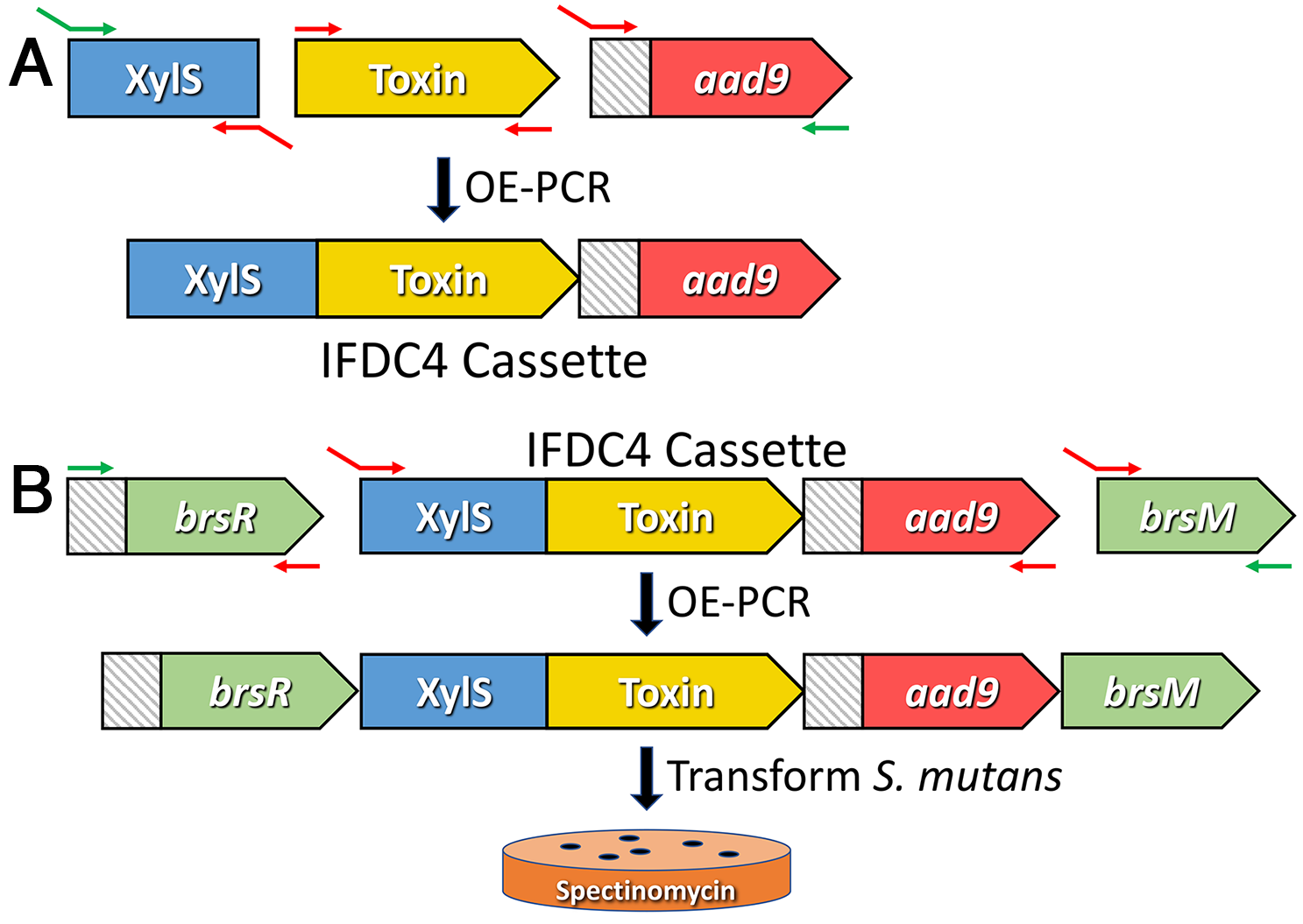

Supplement: FIG S1 [file msphere.00682-22-s0006.tif]
